# 868. HIV, Opioid Use Disorder, and Injection related Infections: Clinical Outcomes in 4 Academic Hospitals

**DOI:** 10.1093/ofid/ofab466.1063

**Published:** 2021-12-04

**Authors:** John R Bassler, Hana Akselrod, Greer A Burkholder, Elana S Rosenthal, Christopher J Brokus, Jillian S Catalanotti, Irene Kuo, Keanan McGonigle, William Mai, Melissa Notis, Kaylee W Burgan, Joseph Carpenter, Alaina Steck, Ellen Eaton, Ellen Eaton

**Affiliations:** 1 University of Alabama at Birmingham, Birmingham, Alabama; 2 The George Washington University of Medicine and Health Sciences, Washington, District of Columbia; 3 University of Maryland, Washington, DC; 4 University of Maryland School of Medicine, Boston, Massachusetts; 5 George Washington University Milken Institute School of Public Health, Washington, DC; 6 University of Alabama Birmingham, Birmingham, Alabama; 7 Emory University School of Medicine, Atlanta, Georgia

## Abstract

**Background:**

Because hospitals are a safety net for persons with injection drug use (IDU), they play a valuable role towards ending the HIV epidemic. The objective of this study is to evaluate the hospital outcomes of persons with HIV (PWH) and opioid use disorder (OUD).

**Methods:**

CHOICE is a retrospective review of hospitalized persons with an infectious complication of OUD and IDU at University of Maryland, George Washington University, University of Alabama at Birmingham, and Grady Memorial Hospital. Participants were hospitalized between 1/2/2018-12/21/2018, had ICD9/10 diagnosis codes consistent with OUD and acute bacterial/fungal infection, and verification of OUD-associated infection. HIV was defined by chart review. We explored HIV viral load (VL), antiretroviral therapy (ART) and medications for opioid use disorder (MOUD) on admission, discharge, consultation, and community care.

Overall CHOICE Study Enrollment

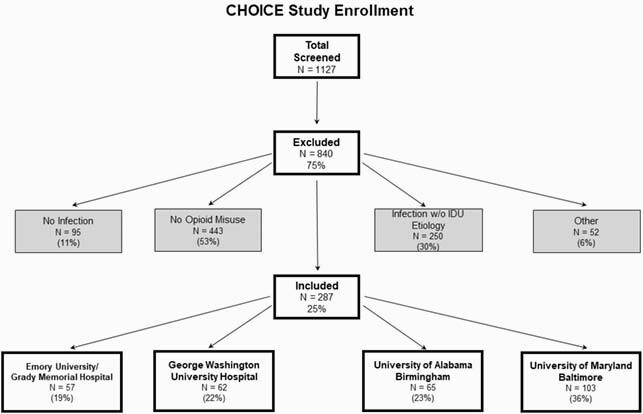

**Results:**

Overall, 287 were admitted with OUD and infections over the study period; 22 had HIV of whom 3 (14%) were diagnosed during the admission. Of the HIV negative, 1 was discharged on PrEP. Of PWH, most were Black (55%), male (68%), and Medicaid recipients (77%); median age was 48. Median length of stay was 10 days. Common bacterial infections were skin/soft tissue (55%), Bacteremia (41%), and Osteomyelitis (18%). On admission, few were on antiretroviral therapy (ART; 32%) or MOUD (23%). Of the 13 with a VL during admission, 100% had viremia (median VL 6,226 copies/mL). During the admission, 81% were evaluated by Infectious Diseases consultant and 50% by Addiction Medicine. At discharge, 11 and 6 had documentation of an ART plan and MOUD receipt, respectively. In the year following the admission, of 21 with follow up data, a majority were evaluated in the emergency department (68%) and readmitted (57%).

HIV Outcomes for Hospitalized Persons with Injection Related Bacterial Infections

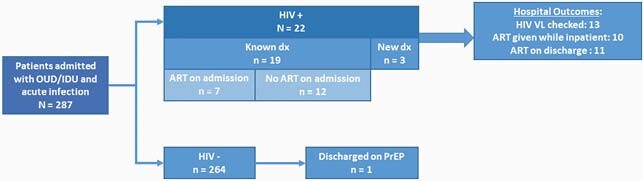

**Conclusion:**

For patients with IDU, hospitalization is a missed opportunity to address HIV treatment and prevention through PrEP, VL surveillance, and ART linkage. Because addiction treatment improves HIV outcomes, Addiction consultation should be standard of care but was under-utilized. Subsequent ED visits and readmissions suggest that hospitals provide continuity of care for patients with IDU who would benefit from HIV, HCV, and other services in acute settings.

**Disclosures:**

**Greer A. Burkholder, MD, MSPH**, **Eli Lilly** (Grant/Research Support) **Elana S. Rosenthal, MD**, **Gilead Sciences** (Research Grant or Support)**Merck** (Research Grant or Support) **Ellen Eaton, MD** , **Gilead** (Grant/Research Support) **Ellen Eaton, MD** , Gilead (Individual(s) Involved: Self): Research Grant or Support

